# Intravenous immunoglobulin for acute hemorrhagic leukoencephalitis refractory to plasmapheresis

**DOI:** 10.1002/ccr3.1843

**Published:** 2018-11-28

**Authors:** Apurva Panchal, Francesca Perez‐Marques

**Affiliations:** ^1^ Department of Pediatrics University of Kansas Medical Center Kansas City Kansas

**Keywords:** acute disseminated encephalomyelitis, acute hemorrhagic leukoencephalitis, autoimmune encephalitis, children, intravenous immunoglobulin, plasmapheresis

## Abstract

Intravenous immunoglobulin therapy should be considered in pediatric acute hemorrhagic leukoencephalitis that is refractory to steroid and plasmapheresis.

## 1 INTRODUCTION

Acute hemorrhagic leukoencephalitis (AHLE) is a rare but an aggressive form of acute disseminated encephalomyelitis (ADEM).^1^, ^2^ It is often associated with cerebral edema, petechial hemorrhage, and necrotizing vasculitis in the brain.^3^, ^4^ These changes frequently affect cerebral white matter, predominantly in the perivascular area.^1^, ^2^, ^3^ Diffuse cerebral edema can rapidly progress to brain herniation, which has a mortality rate of 70%.^1^ The etiology is unclear, but it is thought to be triggered by cross‐reactivity between a viral or bacterial antigen and the patient's myelin protein, leading to autoimmune demyelination.^1^, ^3^ The onset is sudden and the patient usually presents within a few days following a viral or bacterial illness with signs of meningoencephalitis.^3^ Diagnosis can be made with a computed tomography (CT) scan or magnetic resonance imaging (MRI) of the brain.^2^, ^3^ A brain biopsy shows demyelination, hemorrhage, and infiltrates of macrophages, neutrophils and mononuclear cells in perivascular areas.^1^, ^2^, ^3^, ^4^ Prompt diagnosis, vigorous intracranial pressure (ICP) control, and treatment with immunosuppressive therapy and plasmapheresis are associated with better outcomes and increased survival rates.^1^, ^4^

## 2 CASE REPORT

**Figure 1:**
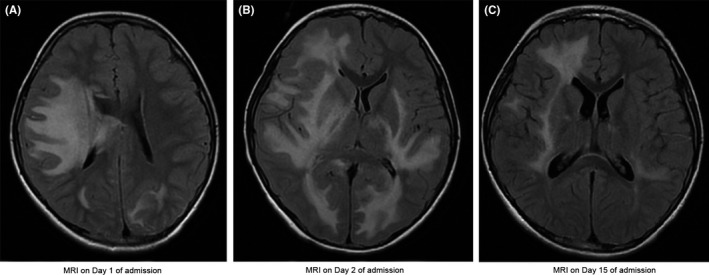
A, MRI (on Day 1 of admission) showing patchy enhancement of cerebral white matter associated with a mild right to left midline shift. B, MRI (on Day 2 of admission) showing a marked progression of diffuse cerebral edema of bilateral white matter, right more than left, with worsening of a midline shift. C, MRI (on Day 15 of admission) showing significant improvement in cerebral edema (less hyperintense white matter area) with a near complete resolution of a midline shift

An 8‐year‐old boy presented to us with a 2‐day history of fever, vomiting, and severe headache. His past medical history included two episodes of concussion and a recent upper respiratory infection. His initial examination was unremarkable. Given the history of head trauma, a head CT scan was obtained and it revealed asymmetrical white matter hypodensity and edema in the right frontal and temporal lobes without evidence of hemorrhage or midline shift, of concern in cases of meningoencephalitis. Cerebrospinal fluid (CSF) was collected and showed marked leukocytosis (1660 cells/L) and increased protein levels (250 mg/dL). The patient was started on ceftriaxone and vancomycin. Within a few hours after admission, the patient's condition deteriorated with left‐sided hemiparesis and altered mental status. His airway was secured with successful intubation. A brain MRI was obtained and showed patchy areas of enhancement throughout the right cerebral white matter, midbrain, and pons with significant fluid‐attenuated inversion recovery (FLAIR) changes—highly suggestive of ADEM. There was minimal right to left midline shift (Figure 1A). Aggressive management of high ICP was initiated due to a concern for cerebral herniation. It included head elevation, hyperosmolar therapy, therapy to maintain euthermia and carbon dioxide level in normal physiological range, sedatives and anti‐epileptic medications to limit brain hyperactivity. The neurosurgical team was consulted for invasive ICP monitoring and for a possible brain biopsy to confirm the diagnosis of ADEM or AHLE. The patient was started on a pulse dose of intravenous methylprednisolone of 30 mg/kg/d for 5 days. On the second day of admission, the patient started posturing with further deterioration. A repeat MRI showed worsening of the midline shift with early tonsillar herniation secondary to massive edema, now involving both hemispheres (Figure 1B). The MRI further showed a new hemorrhage in the right cerebral hemisphere. Based on the rapid deterioration of his clinical status and new findings of hemorrhage with widespread white matter cerebral edema on the MRI, the diagnosis of AHLE was clinically made. An external ventricular drain (EVD) was put in place and the patient started on plasmapheresis (volume 1.0). Plasmapheresis was continued for 5 days without notable clinical improvement. Subsequently, the patient was given a dose of intravenous immunoglobulin (IVIG) 2 g/kg for two days. A dramatic improvement in his mentation, neurological deficits, and ICP followed over the next few days. The EVD and endotracheal tube were removed, and the patient weaned off sedative medications and hyperosmolar therapy. A repeat MRI on day 15 post‐admission showed marked improvement in the cerebral edema with a complete resolution of the midline shift and tonsillar herniation (Figure 1C). He had an extensive infectious disease workup that was entirely negative (Table 1). On hospital day 20, the patient was transferred to a rehabilitation facility with levetiracetam and a tapering dose of prednisolone.

**Table 1:** Common pathogens associated with AHLE

Viruses	Herpes simplex virus
	Cytomegalovirus
	Epstein‐Barr virus
	Human herpesvirus 6
	Varicella zoster virus
	Respiratory syncytial virus
	Influenza
	John Cunningham virus
	West Nile
	California
	Eastern and western equine viruses
Bacteria	Mycoplasma
	Borrelia burgdorferi
	Streptococcus
	Leptospira
	Chlamydia
	Rickettsia

## 3 DISCUSSION

Acute hemorrhagic leukoencephalitis, also known as Hurst disease, after Dr E. Weston Hurst who first described it in 1941, is a fatal form of ADEM.^3^ It is characterized by diffuse inflammation and hemorrhagic necrosis of white matter predominantly around the small parenchymal blood vessels.^1^, ^3^, ^4^ The etiology of AHLE is unclear, but studies suggest it results from the autoimmune destruction of white matter that occurs secondary to a cross‐reactivity phenomenon following a viral or bacterial illness.^1^, ^3^ Various viruses and bacteria (Table 1) have been associated with ADEM/AHLE.^1^, ^3^, ^4^ Tenembaum found a genetic predisposition for AHLE in patients with particular major histocompatibility complex (MHC) haplotypes.^5^

Patients with AHLE present abruptly (within a few days of illness onset) with fever, headache, meningismus, seizure, or focal neurological deficit.^3^ The differential diagnosis includes meningoencephalitis, ADEM, stroke, autoimmune encephalitis or vasculitis, multiple sclerosis, and leukodystrophies.^2^, ^3^ Diagnosis can be made clinically and, more importantly, by CSF studies, CT scans, MRIs, and a brain biopsy.^2^, ^3^ CSF studies are typically positive for leukocytosis and increased protein levels, and negative for cultures and for polymerase chain reaction testing for infectious causes.^2^, ^3^ A head CT scan may show hypodensities in affected white and gray matter.^3^ A brain MRI will show hyperintense T2‐weighted lesions. The FLAIR sequence will provide better visualization.^2^, ^3^ A brain biopsy may reveal the pathognomonic findings of demyelination, hemorrhagic necrosis, and perivascular infiltrates.^1^, ^3^ While direct brain tissue examination provides a definitive diagnosis,^3^ it may not be feasible in all patients with suspected AHLE. The diagnosis can be made clinically based on symptom severity, clinical course of the disease, and radiological findings.^2^ ADEM and AHLE share radiological findings, but AHLE typically has extensive cerebral edema and it frequently presents with intracranial hemorrhage.^6^

Acute hemorrhagic leukoencephalitis carries a poor prognosis, with death typically occurring within a week of onset due to increased ICP.^1^, ^2^, ^3^, ^4^ Treatment of AHLE can change the fulminant course of the disease.^1^, ^4^ Treatment first includes aggressive control of ICP, which sometimes requires decompressive craniectomy.^1^ However, most patients can be managed medically. The second part of treatment consists of immunomodulation therapy to address the ongoing intense inflammation. There are no established treatment guidelines for AHLE.^3^, ^4^ but multiple case reports and small studies support the use of high‐dose steroids, IVIG, and plasmapheresis as the mainstay treatment for AHLE.^2^, ^3^, ^4^ Ideally, a pulse dose of IV methylprednisolone should be started on initial presentation along with antibiotics and acyclovir. It should be continued for 3‐5 days followed by oral prednisolone for 4‐6 weeks.^5^ Various case reports and small studies have demonstrated the effectiveness of daily IVIG therapy either as a single dose or as a five‐day course.^7^ Although no therapeutic trials comparing IVIG and plasmapheresis for the management of AHLE exist, the evidence from numerous case reports and series emphasizes the use of plasmapheresis when steroid and IVIG fail.^4^, ^8^ Due to a concern that plasmapheresis administered immediately after IVIG would decrease effectiveness of IVIG, we decided to try IVIG after plasmapheresis only if the latter failed to improve the condition. To our surprise, the patient's condition improved dramatically after two doses of IVIG therapy with a slow return of mental and neurological status over next few days. To our knowledge, this is the first case of plasmapheresis‐refractory pediatric AHLE being successfully treated with IVIG without significant morbidity.

In conclusion, AHLE should be considered in patients with suspected ADEM who show rapid clinical deterioration. Treatment should include pulse dose steroid and plasmapheresis. We recommend considering IVIG if plasmapheresis is ineffective to treat AHLE.

## CONFLICT OF INTEREST

All the authors declared no conflict of interest.

## AUTHOR CONTRIBUTION

All authors: contributed toward the case report by making the substantial contribution. Drs. AP and FPM: participated in the medical care of this patient and in the preparation of the manuscript.
